# (1*S*,5*R*)-1-(4-Fluoro­phen­yl)-3-azonia­bicyclo­[3.1.0]hexane chloride

**DOI:** 10.1107/S1600536809006953

**Published:** 2009-03-06

**Authors:** Carl Henrik Görbitz, Tore Hansen, Kristian Vestli

**Affiliations:** a Department of Chemistry, University of Oslo, PO Box 1033 Blindern, N-0315 Oslo, Norway

## Abstract

The absolute structure of the title compound, C_11_H_13_FN^+^·Cl^−^, has been determined. The five-membered ring has an envelope conformation with the N atom at the flap position. In the crystal structure, the Cl^−^ anion links with the organic cation *via* N—H⋯Cl hydrogen bonding.

## Related literature

For related structures, see: McArdle *et al.* (2004 ▶).
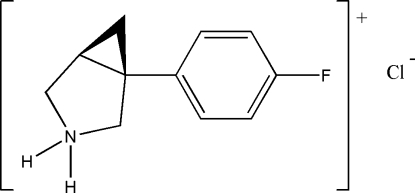


## Experimental

### 

#### Crystal data

C_11_H_13_FN^+^·Cl^−^
*M* *_r_* = 213.67
Orthorhombic, 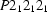

*a* = 6.9146 (10) Å
*b* = 7.8048 (11) Å
*c* = 19.448 (3) Å
*V* = 1049.6 (3) Å^3^
*Z* = 4
Mo *K*α radiation
μ = 0.34 mm^−1^
*T* = 296 K
0.50 × 0.36 × 0.25 mm

#### Data collection

Bruker APEXII CCD diffractometer
Absorption correction: multi-scan (*SADABS*; Sheldrick, 1996 ▶) *T*_min_ = 0.766, *T*_max_ = 0.919
6726 measured reflections
2292 independent reflections
2244 reflections with *I* > 2σ(*I*)
*R*_int_ = 0.028

#### Refinement

*R*[*F* ^2^ > 2σ(*F* ^2^)] = 0.025
*wR*(*F* ^2^) = 0.066
*S* = 1.05
2292 reflections
133 parameters
H atoms treated by a mixture of independent and constrained refinement
Δρ_max_ = 0.38 e Å^−3^
Δρ_min_ = −0.17 e Å^−3^
Absolute structure: Flack (1983 ▶), 934 Friedel pairs
Flack parameter: −0.03 (5)

### 

Data collection: *APEX2* (Bruker, 2007 ▶); cell refinement: *SAINT-Plus* (Bruker, 2007 ▶); data reduction: *SAINT-Plus*; program(s) used to solve structure: *SHELXTL* (Sheldrick, 2008 ▶); program(s) used to refine structure: *SHELXTL*; molecular graphics: *SHELXTL*; software used to prepare material for publication: *SHELXTL*.

## Supplementary Material

Crystal structure: contains datablocks I, global. DOI: 10.1107/S1600536809006953/xu2479sup1.cif

Structure factors: contains datablocks I. DOI: 10.1107/S1600536809006953/xu2479Isup2.hkl

Additional supplementary materials:  crystallographic information; 3D view; checkCIF report

## Figures and Tables

**Table 1:** Hydrogen-bond geometry (Å, °)

*D*—H⋯*A*	*D*—H	H⋯*A*	*D*⋯*A*	*D*—H⋯*A*
N1—H1⋯Cl1	0.939 (18)	2.146 (18)	3.0837 (13)	176.1 (15)
N1—H2⋯Cl1^i^	0.899 (17)	2.275 (17)	3.0907 (13)	150.8 (14)

Symmetry code: (i) 
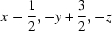
.

**Fig. 1.**
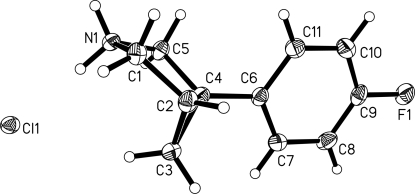
The asymmetric unit of (I). Displacement ellipsoids are shown at the 50% probability level and H atoms are shown as spheres of arbitrary size.

## supplementary crystallographic information

### Comment

The title compound was prepared as a potential triple neurotransmittor reuptake inhibitor. Details will be published elsewhere. The molecular structure is shown in Fig. 1. The five-membered ring has an envelope conformation with N1 located 0.454 (2) Å above the plane constituted by C1, C2, C4 and C5, on the same side as C3, giving the six-membered ring N1—C1—C2—C3—C4—C5 a distinct boat conformation.

Two different *P*2_1_/c polymorphs, with *Z*' = 1 and 4, respectively, were obtained for the racemate of bicifadine hydrochloride (McArdle *et al.*, 2004), which has a methyl group rather than a F atom in the phenyl ortho position. Ring puckering remains unchanged, but phenyl rotations vary; C5—C4—C6—C11 is thus 59.81 (19)° for the title compound, but 83.9° for polymorph 1 of bicifadine and between -10.1 and -30.8° for polymorph 2 (1*S*,5*R*-enatiomers).

### Experimental

Block-shaped crystals were prepared from an acetonitrile solution by slow evaporation at room temperature.

### Refinement

Positional parameters were refined for the two H atoms bonded to N. Other H atoms were positioned with idealized geometry and fixed C—H = 0.93 (aromatic), 0.97 (methylene) or 0.98 Å (methine). *U*_iso_(H) values were 1.2*U*_eq_(C,N).

### Crystal data

**Table:**

C_11_H_13_FN^+^·Cl^−^	*D*_x_ = 1.352 Mg m^−^^3^
*M**_r_* = 213.67	Mo *K*α radiation, λ = 0.71073 Å
Orthorhombic, *P*2_1_2_1_2_1_	Cell parameters from 4938 reflections
*a* = 6.9146 (10) Å	θ = 2.1–27.1°
*b* = 7.8048 (11) Å	µ = 0.34 mm^−^^1^
*c* = 19.448 (3) Å	*T* = 296 K
*V* = 1049.6 (3) Å^3^	Block, colourless
*Z* = 4	0.50 × 0.36 × 0.25 mm
*F*(000) = 448	

### Data collection

**Table:**

Bruker APEXII CCD diffractometer	2292 independent reflections
Radiation source: fine-focus sealed tube	2244 reflections with *I* > 2σ(*I*)
graphite	*R*_int_ = 0.028
Detector resolution: 8.3 pixels mm^-1^	θ_max_ = 27.1°, θ_min_ = 2.1°
sets of exposures each taken over 0.5° ω rotation scans	*h* = −8→4
Absorption correction: multi-scan (*SADABS*; Sheldrick, 1996)	*k* = −9→10
*T*_min_ = 0.766, *T*_max_ = 0.919	*l* = −24→24
6726 measured reflections	

### Refinement

**Table:**

Refinement on *F*^2^	Secondary atom site location: difference Fourier map
Least-squares matrix: full	Hydrogen site location: inferred from neighbouring sites
*R*[*F*^2^ > 2σ(*F*^2^)] = 0.025	H atoms treated by a mixture of independent and constrained refinement
*wR*(*F*^2^) = 0.066	*w* = 1/[σ^2^(*F*_o_^2^) + (0.036*P*)^2^ + 0.1918*P*] where *P* = (*F*_o_^2^ + 2*F*_c_^2^)/3
*S* = 1.05	(Δ/σ)_max_ < 0.001
2292 reflections	Δρ_max_ = 0.38 e Å^−^^3^
133 parameters	Δρ_min_ = −0.17 e Å^−^^3^
0 restraints	Absolute structure: Flack (1983), 934 Friedel pairs
Primary atom site location: structure-invariant direct methods	Flack parameter: −0.03 (5)

### Special details

**Table:**

Experimental. Crystallized from acetonitrile solution
Geometry. All e.s.d.'s (except the e.s.d. in the dihedral angle between two l.s. planes) are estimated using the full covariance matrix. The cell e.s.d.'s are taken into account individually in the estimation of e.s.d.'s in distances, angles and torsion angles; correlations between e.s.d.'s in cell parameters are only used when they are defined by crystal symmetry. An approximate (isotropic) treatment of cell e.s.d.'s is used for estimating e.s.d.'s involving l.s. planes.
Refinement. Data were collected by measuring three sets of exposures with the detector set at 2θ = 29°, crystal-to-detector distance 6.00 cm. Refinement of *F*^2^ against ALL reflections.

### Fractional atomic coordinates and isotropic or equivalent isotropic displacement parameters (Å^2^)

**Table:**

	*x*	*y*	*z*	*U*_iso_*/*U*_eq_	
Cl1	0.90409 (5)	0.57733 (4)	0.024884 (16)	0.01896 (9)	
F1	−0.08580 (14)	0.45054 (11)	0.37784 (4)	0.0280 (2)	
C6	0.2562 (2)	0.43164 (19)	0.20420 (7)	0.0184 (3)	
C5	0.3941 (2)	0.60246 (17)	0.10328 (6)	0.0185 (3)	
H51	0.2695	0.6595	0.1020	0.022*	
H52	0.4871	0.6774	0.1254	0.022*	
N1	0.45923 (17)	0.55635 (15)	0.03221 (6)	0.0168 (2)	
H1	0.595 (3)	0.557 (2)	0.0300 (8)	0.020*	
H2	0.422 (3)	0.640 (2)	0.0035 (8)	0.020*	
C4	0.3797 (2)	0.43318 (18)	0.14105 (6)	0.0172 (3)	
C7	0.3340 (2)	0.3976 (2)	0.26874 (7)	0.0225 (3)	
H71	0.4644	0.3701	0.2726	0.027*	
C11	0.0603 (2)	0.4731 (2)	0.19938 (7)	0.0235 (3)	
H111	0.0067	0.4972	0.1566	0.028*	
C9	0.0279 (2)	0.44386 (19)	0.32031 (7)	0.0217 (3)	
C1	0.3826 (2)	0.38066 (16)	0.01663 (7)	0.0190 (3)	
H11	0.4678	0.3197	−0.0145	0.023*	
H12	0.2545	0.3868	−0.0036	0.023*	
C8	0.2196 (2)	0.4041 (2)	0.32777 (7)	0.0248 (3)	
H81	0.2720	0.3821	0.3709	0.030*	
C2	0.3764 (2)	0.29489 (18)	0.08635 (7)	0.0206 (3)	
H21	0.2921	0.1953	0.0929	0.025*	
C3	0.5512 (2)	0.31501 (19)	0.13212 (7)	0.0224 (3)	
H31	0.6677	0.3624	0.1119	0.027*	
H32	0.5730	0.2285	0.1671	0.027*	
C10	−0.0560 (2)	0.4790 (2)	0.25780 (8)	0.0255 (3)	
H101	−0.1867	0.5059	0.2546	0.031*	

### Atomic displacement parameters (Å^2^)

**Table:**

	*U*^11^	*U*^22^	*U*^33^	*U*^12^	*U*^13^	*U*^23^
Cl1	0.01835 (16)	0.01641 (15)	0.02212 (15)	−0.00080 (13)	−0.00166 (13)	0.00305 (12)
F1	0.0339 (5)	0.0273 (5)	0.0227 (4)	0.0066 (4)	0.0116 (4)	0.0024 (3)
C6	0.0212 (7)	0.0151 (6)	0.0189 (6)	−0.0006 (6)	0.0024 (5)	0.0012 (5)
C5	0.0235 (7)	0.0150 (6)	0.0170 (6)	−0.0025 (6)	0.0013 (6)	0.0003 (5)
N1	0.0187 (6)	0.0147 (5)	0.0169 (5)	0.0003 (5)	−0.0001 (4)	0.0017 (4)
C4	0.0182 (6)	0.0161 (6)	0.0172 (6)	0.0000 (6)	0.0002 (5)	0.0013 (5)
C7	0.0215 (7)	0.0241 (7)	0.0217 (6)	0.0035 (6)	0.0007 (5)	0.0037 (6)
C11	0.0230 (8)	0.0292 (7)	0.0184 (6)	−0.0007 (6)	−0.0016 (5)	0.0019 (5)
C9	0.0292 (7)	0.0160 (7)	0.0198 (6)	0.0007 (6)	0.0086 (6)	0.0005 (5)
C1	0.0209 (7)	0.0162 (6)	0.0198 (6)	−0.0026 (5)	0.0019 (6)	−0.0017 (5)
C8	0.0318 (8)	0.0253 (7)	0.0174 (6)	0.0046 (7)	0.0003 (6)	0.0049 (6)
C2	0.0250 (7)	0.0137 (6)	0.0232 (6)	−0.0006 (6)	0.0044 (6)	0.0004 (5)
C3	0.0227 (7)	0.0230 (7)	0.0216 (7)	0.0055 (6)	0.0024 (5)	0.0066 (5)
C10	0.0204 (7)	0.0302 (8)	0.0259 (7)	0.0024 (6)	0.0025 (6)	0.0016 (6)

### Geometric parameters (Å, °)

**Table:**

F1—C9	1.3683 (15)	C7—H71	0.9300
C6—C7	1.3911 (19)	C11—C10	1.393 (2)
C6—C11	1.396 (2)	C11—H111	0.9300
C6—C4	1.4959 (18)	C9—C8	1.369 (2)
C5—N1	1.4977 (16)	C9—C10	1.375 (2)
C5—C4	1.5149 (18)	C1—C2	1.5128 (18)
C5—H51	0.9700	C1—H11	0.9700
C5—H52	0.9700	C1—H12	0.9700
N1—C1	1.5011 (16)	C8—H81	0.9300
N1—H1	0.939 (18)	C2—C3	1.509 (2)
N1—H2	0.899 (17)	C2—H21	0.9800
C4—C3	1.512 (2)	C3—H31	0.9700
C4—C2	1.5156 (19)	C3—H32	0.9700
C7—C8	1.395 (2)	C10—H101	0.9300
			
C7—C6—C11	118.70 (12)	F1—C9—C8	118.57 (13)
C7—C6—C4	121.43 (13)	F1—C9—C10	118.25 (13)
C11—C6—C4	119.80 (12)	C8—C9—C10	123.18 (13)
N1—C5—C4	104.93 (10)	N1—C1—C2	103.48 (11)
N1—C5—H51	110.8	N1—C1—H11	111.1
C4—C5—H51	110.8	C2—C1—H11	111.1
N1—C5—H52	110.8	N1—C1—H12	111.1
C4—C5—H52	110.8	C2—C1—H12	111.1
H51—C5—H52	108.8	H11—C1—H12	109.0
C5—N1—C1	107.42 (10)	C9—C8—C7	118.03 (13)
C5—N1—H1	109.9 (9)	C9—C8—H81	121.0
C1—N1—H1	110.4 (10)	C7—C8—H81	121.0
C5—N1—H2	108.2 (11)	C3—C2—C1	117.40 (13)
C1—N1—H2	116.1 (11)	C3—C2—C4	59.99 (9)
H1—N1—H2	104.7 (16)	C1—C2—C4	108.28 (11)
C3—C4—C2	59.78 (10)	C3—C2—H21	118.9
C3—C4—C5	115.15 (12)	C1—C2—H21	118.9
C3—C4—C6	122.49 (12)	C4—C2—H21	118.9
C6—C4—C5	116.26 (12)	C2—C3—C4	60.23 (9)
C6—C4—C2	124.19 (12)	C2—C3—H31	117.7
C5—C4—C2	106.36 (10)	C4—C3—H31	117.7
C6—C7—C8	121.09 (14)	C2—C3—H32	117.7
C6—C7—H71	119.5	C4—C3—H32	117.7
C8—C7—H71	119.5	H31—C3—H32	114.9
C10—C11—C6	120.85 (13)	C9—C10—C11	118.15 (14)
C10—C11—H111	119.6	C9—C10—H101	120.9
C6—C11—H111	119.6	C11—C10—H101	120.9
			
C1—C2—C4—C5	−1.68 (16)	C5—N1—C1—C2	−30.53 (14)
N1—C1—C2—C4	19.46 (15)	F1—C9—C8—C7	179.97 (13)
N1—C5—C4—C2	−16.93 (15)	C10—C9—C8—C7	−0.6 (3)
C5—C4—C6—C11	59.81 (19)	C6—C7—C8—C9	0.4 (2)
C4—C5—N1—C1	29.91 (15)	N1—C1—C2—C3	−45.58 (15)
C7—C6—C4—C3	34.1 (2)	C6—C4—C2—C3	−110.84 (15)
C11—C6—C4—C3	−149.04 (14)	C5—C4—C2—C3	109.97 (13)
C7—C6—C4—C2	107.32 (17)	C6—C4—C2—C1	137.51 (14)
C11—C6—C4—C2	−75.82 (19)	C3—C4—C2—C1	−111.65 (14)
C7—C6—C4—C5	−117.04 (15)	C1—C2—C3—C4	96.23 (13)
N1—C5—C4—C6	−159.86 (12)	C6—C4—C3—C2	113.59 (15)
N1—C5—C4—C3	46.86 (15)	C5—C4—C3—C2	−94.97 (12)
C11—C6—C7—C8	0.2 (2)	F1—C9—C10—C11	179.61 (13)
C4—C6—C7—C8	177.04 (14)	C8—C9—C10—C11	0.2 (2)
C7—C6—C11—C10	−0.6 (2)	C6—C11—C10—C9	0.4 (2)
C4—C6—C11—C10	−177.53 (14)		

### Hydrogen-bond geometry (Å, °)

**Table:**

*D*—H···*A*	*D*—H	H···*A*	*D*···*A*	*D*—H···*A*
N1—H1···Cl1	0.939 (18)	2.146 (18)	3.0837 (13)	176.1 (15)
N1—H2···Cl1^i^	0.899 (17)	2.275 (17)	3.0907 (13)	150.8 (14)

Symmetry codes: (i) *x*−1/2, −*y*+3/2, −*z*.
